# Maintenance of memory-type pathogenic Th2 cells in the pathophysiology of chronic airway inflammation

**DOI:** 10.1186/s41232-018-0067-8

**Published:** 2018-06-20

**Authors:** Kiyoshi Hirahara, Kenta Shinoda, Yusuke Endo, Tomomi Ichikawa, Toshinori Nakayama

**Affiliations:** 1 Department of Immunology, Graduate School of Medicine, Chiba University, 1-8-1 Inohana, Chuo-ku, Chiba-shi, Chiba, 260-8670 Japan; 2 AMED-CREST, AMED, 1-8-1 Inohana Chuo-ku, Chiba, 260-8670 Japan

**Keywords:** Memory-type pathogenic Th2 cells, Interleukin-33, Inducible bronchus-associated lymphoid tissue (iBALT), Chronic inflammation

## Abstract

**Background:**

Immunological memory is critical for long-standing protection against microorganisms; however, certain antigen-specific memory CD4^+^ T helper (Th) cells drive immune-related pathology, including chronic allergic inflammation such as asthma. The IL-5-producing memory-type Tpath2 subset is important for the pathogenesis of chronic allergic inflammation. This memory-type pathogenic Th2 cell population (Tpath2) can be detected in various allergic inflammatory lesions. However, how these pathogenic populations are maintained at the local inflammatory site has remained unclear.

**Methods:**

We performed a series of experiments using mice model for chronic airway inflammation. We also investigated the human samples from patients with eosinophilic chronic rhinosinusitis.

**Results:**

We recently reported that inducible bronchus-associated lymphoid tissue (iBALT) was shaped during chronic inflammation in the lung. We also found that memory-type Tpath2 cells are maintained within iBALT. The maintenance of the Tpath2 cells within iBALT is supported by specific cell subpopulations within the lung. Furthermore, ectopic lymphoid structures consisting of memory CD4^+^ T cells were found in nasal polyps of eosinophilic chronic rhinosinusitis patients, indicating that the persistence of inflammation is controlled by these structures.

**Conclusion:**

Thus, the cell components that organize iBALT formation may be therapeutic targets for chronic allergic airway inflammation.

## Background

Asthma is characterized by chronic airway inflammation, mucus hyperproduction, airway hyperresponsiveness, and variable airway obstruction. The pathophysiology of chronic airway inflammation involves in various types of immune cells such as CD4^+^ T cells, B cells, innate lymphoid cells, and eosinophils. In particular, T helper (Th) 2 cells and type 2 innate lymphoid cells play central roles in the pathogenesis of allergic airway inflammation.

**Fig. 1:**
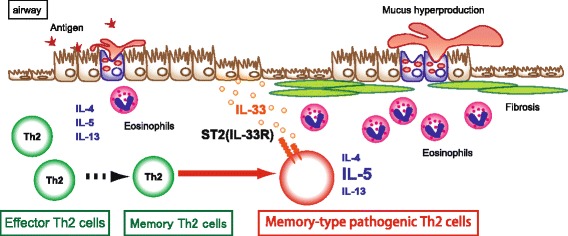
Induction of memory-type pathogenic Th2 cells. IL-33 stimulation induced IL-5-producing memory-type pathogenic Th2 cells at the local inflammatory site

Recent studies have identified “epithelial cytokines” such as IL-25, IL-33, and TSLP as key modulators of type 2 immune responses. IL-33 is constitutively expressed on epithelial cells in mucosal barrier organs [1]. Chronic repeated exposure to various exogenous allergens or pathogens, such as tobacco smoke or inhaled irritant particles, prompts epithelial cells to release their stored IL-33, which is involved in chronic allergic inflammatory diseases such as asthma, eosinophilic chronic rhinosinusitis (ECRS), pollen allergy, and eosinophilic pneumonia. IL-33 was originally identified as a ligand for the ST2 receptor (also known as IL1RL1) [2]. Effector Th2 cells, regulatory T cells, mast cells, and ILC2s are known to be target cells of IL-33. We found that memory-type pathogenic Th2 (Tpath2) cells, which produce large amounts of IL-5, expressed high levels of ST2 [3, 4] (Fig. 1). The expression of ST2 on memory-type Tpath2 cells was higher than that on effector Th2 cells, which suggested that memory-type Tpath2 cells were novel targets of IL-33 in vivo.

In humans, it has been shown that bronchus-associated lymphoid tissue (BALT) is shaped in the lung in response to inflammatory states caused by infectious organisms, smoking, and auto-immune diseases; under these conditions, this tissue known as inducible BALT (iBALT) [5, 6]. For example, patients with chronic obstructive pulmonary disease (COPD) showed ectopic lymphoid structures in the lungs [5]. However, whether or not iBALT is involved in the pathophysiology of chronic allergic diseases, such as asthma, and how memory-type T cells are maintained in the local inflammatory tissues has been unclear.

## Results

**Fig. 2:**
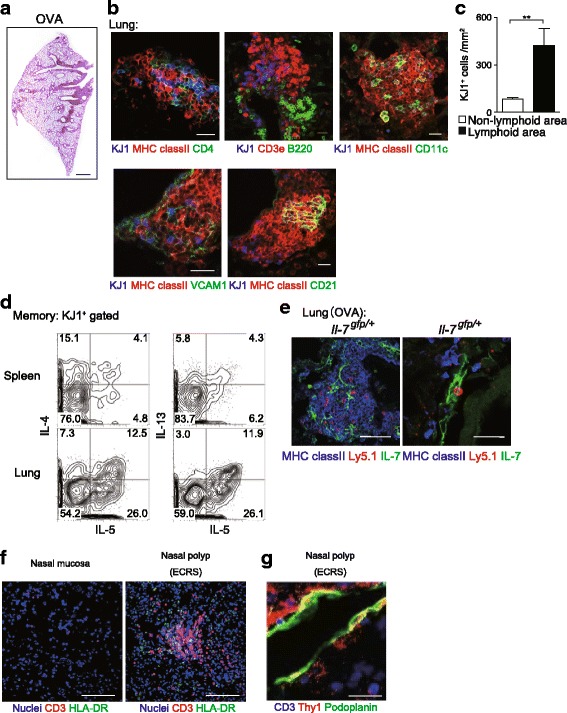
iBALT is induced under conditions of chronic allergic inflammation in both mice and humans. **a** The intra-nasal administration of the antigen resulted in iBALT formation in the lungs of mice. **b** iBALT included memory Th2 cells, MHC-class ll^+^ cells, B220^+^ cells, CD11c^+^ cells, VCAM1^+^ cells, and CD21^+^ cells. **c** The memory Th2 cells showed greater accumulation in the lymphoid areas than in the non-lymphoid areas. **d** Memory Th2 cells in the lung produced more Th2 cytokines compared to those from memory Th2 cells in the spleen. **e** IL-7-producing cells and Ly5.1^+^ memory Th2 cells were detected in mice iBALT. **f** Ectopic lymphoid structures were generated in the polyps of patients with ECRS. **g** Podoplanin-positive lymphatics were increased in the polyps of patients with ECRS. (*Shinoda* et al. *PNAS*(2016) Copyright (2016) National Academy of Sciences). KJ: OVA-specific T cell receptor

To determine whether or not iBALT was induced in chronic allergic inflammation, we generated OVA-specific effector Th2 cells in vitro and then adoptively transferred them to syngeneic mice that were intra-nasally administered OVA twice. We analyzed these mice 42 days after the adoptive transfer. Hematoxylin and eosin (HE) staining of the mouse lungs showed that massive infiltration of inflammatory cells had been induced and persisted even 42 days after the intra-nasal administration of OVA (Fig. 2a). Immuno-histochemical staining showed the formation of iBALT structures containing donor-derived memory Th2 cells that were detected by KJ-1.26 (KJ1), which is a monoclonal antibody that recognizes OVA-specific TCR DO11.10, MHC class-ll-positive cells, B220-positive cells, CD11c-positive cells, stromal cells, and CD21-positive follicular dendritic cells (Fig. 2b). These results indicate that the iBALT detected in our experimental model was comparable to that noted in previous reports [6]. Notably, the memory Th2 cells showed greater accumulation in iBALT than in non-lymphoid areas (Fig. 2c).

We noted no difference in the number of memory Th2 cells in the spleen with and without intranasal administration of OVA. In sharp contrast, we observed a significant increase in the number of memory Th2 cells in the lung following the intranasal administration of OVA. These memory Th2 cells in the lung produced increased levels of IL-5 (Fig. 2d). Taken together, these findings show that the adoptive transfer of effector Th2 cells followed by the intra-nasal administration of OVA resulted in iBALT formation and the accumulation of memory-type Tpath2 cells in the lung.

We then assessed the patho-physiological role of memory-type Tpath2 cells maintained in iBALT. The OVA-induced airway inflammatory responses were assessed using the mice with iBALT formation. iBALT-induced mice showed enhanced infiltration of inflammatory cells in the BALF compared with the control animals. Consistent with this result, the airway hyperresponsiveness and mucus production were enhanced in the mice with iBALT. Thus, the memory-type Tpath2 cells in the mice with iBALT were involved in the pathogenicity of eosinophilic airway inflammation.

IL-7 is a key cytokine involved in the maintenance of T cells in vivo [7]. We therefore wanted to determine whether or not IL-7 was involved in the maintenance of memory Th2 cells in iBALT using IL-7 GFP knock-in mice (collaboration with Professor Ikuta in Kyoto University). We found that a main population of IL-7-producing cells was accumulated in iBALT in the lung (Fig. 2e). Within the iBALT, most memory Th2 cells were co-localized with IL-7-producing cells. When we analyzed the PECAM-1-positive endothelial cells, *Pdpn* and *Prox1*, which are specific markers for lymphatic endothelial cells, were highly expressed in the isolated PECAM1^+^IL-7-GFP^+^ cells. Interestingly, PECAM1^+^IL-7-GFP^+^ cells also expressed *Il33* mRNA. A FACS analysis revealed that the PECAM1^+^IL-7-GFP^+^ cells expressed Lyve-1 and podoplanin. Taken together, these results suggest that lympathic endothelial cells in iBALT produce IL-7. We also found that PECAM1^+^IL-7-GFP^+^ cells showed high expression of Thy1. We generated *Il-7*^*flox/flox*^ mice crossed with *Tie2*-Cre transgenic mice, in which the mouse endothelial-specific receptor tyrosine kinase (Tie2) promoter directs expression of Cre recombinase, to investigate the role of IL-7 produced by LECs. When iBALT was induced using *Il-7*^fl/fl^*Tie2-Cre*^+^ Tg mice as hosts, iBALT formation was impaired in the lung of *Il-7*^fl/fl^*Tie2-Cre*^+^ Tg mice. We also detected decreased numbers of memory Th2 cells in the lung. Taken together, these findings indicate that Thy1^+^IL-7^+^ lymphatic endothelial cells (LECs) support the memory Th2 cell survival in iBALT in vivo.

IL-5-producing Tpath2 cells have been detected in the PBMCs of patients with eosinophilic gastrointestinal disease [8]. However, whether or not Tpath2 cells are maintained in the local inflammatory tissue in humans has been unclear. ECRS is a chronic upper respiratory airway allergic disease characterized by the formation of nasal polyps and the infiltration of massive eosinophils in the polyps [9]. We analyzed local inflammatory tissues from the polyps of patients with ECRS. Very little T cell infiltration and few lymphoid structures were detected in the nasal mucosa of control subjects. However, in sharp contrast, the nasal polyps of patients with ECRS showed massive infiltration of CD3^+^ T cells accompanied by elevated numbers of ectopic lymphoid structures (Fig. 2f). The majority of accumulated CD3^+^ T cells were memory-type CD4^+^ T cells, as they expressed CD4 together with CD45RO. Furthermore, podoplanin-positive lymphatics were increased in the nasal polyps of patients with ECRS compared to the control nasal mucosa (Fig. 2g). *IL7* and *IL33* were expressed more strongly in CD45^−^PECAM1^+^Thy1^+^ cells than in CD45^−^PECAM1^+^Thy1^−^ cells.

## Discussion

Our research highlighted that Thy1^+^IL-7^+^ lymphatic endothelial cells (LECs) support memory Th2 cell survival in iBALT in the chronic inflamed lung from mice [10]. Moreover, we found that memory-type CD4^+^ T cells and IL-7^+^IL-33^+^ LECs accumulated in polyps from ECRS patients. These results indicate that Thy1^+^IL-7^+^ LECs produce IL-33 and may confer the pathogenicity on Tpath2 cells. The major IL-7-producing cells in the iBALT are the LECs that are co-localized with memory Th2 cells in the lung. A set of experiments by using IL-7 conditional knockout mice (*Tie2*-Cre^+^*Il-7*^fl/fl^ mice) verified the importance of IL-7-production from LECs on the maintenance of memory Th2 cells in iBALT. Thus, these cells likely provide a survival niche for memory Th2 cells at local inflammatory sites in the airway. Further study is needed to investigate the contribution of IL-7 to T cell-mediated chronic inflammatory diseases such as steroid-resistant asthma.

## Conclusion

In summary, our findings showed that the iBALT structure supports the Tpath2 cell survival in chronic airway inflammation. The cell components and or functional molecules that organize iBALT formation may be therapeutic targets for chronic allergic airway inflammation.

## Abbreviations

BALF: Bronchoalveolar lavage fluid
COPD: Chronic obstructive pulmonary disease
iBALT: Inducible bronchus-associated lymphoid tissue
IL: Interleukin
ILC2s: Type 2 innate lymphoid cells
Tpath2 cells: Pathogenic Th2 cells
